# Applying a Program Increasing Empathy in Expectant Couples to Adolescent Mental Health Promotion

**DOI:** 10.31662/jmaj.2021-0162

**Published:** 2021-09-27

**Authors:** Noriko Kato

**Affiliations:** 1 Department of Early Child Care and Education, Jumonji University, Niiza, Japan

**Keywords:** empathy, school health, adolescent

The final evaluation of the Healthy Parents and Children 21 Campaign in 2014 identified the suicide rate among teens as one of the two parameters that are difficult to improve. The second stage of this campaign focuses on health measures for school-age children/adolescents and young adults as a fundamental challenge. The suicide rate per 100,000 population among young people aged 15-19 was 7.5, 7.2, and 9.9 in 2010, 2016, and 2019, respectively. Addressing mental health problems faced by young adults may be a challenge for Japan to urgently address. Bullying deeply reflects adolescent mental health issues, and it has serious impacts, resulting in mental and physical health problems, chronic absence from school, low self-esteem, and suicide. Deterioration in mental health during adolescence does not only make young people themselves suffer but may also lead to pregnancy at an unexpected point in the future or high-risk pregnancy. It may also cause postpartum depression or other mental health disorders during the perinatal period and even child abuse.

The World Health Organization (WHO) defines the abilities and skills needed to constructively and effectively manage various problems and needs that arise in daily life as “life skills.” Regarding reproductive health education and life skills education as effective health education for adolescents, it recommends education programs to acquire ten life skills, including empathy, communication, interpersonal relationship-building, and stress management ^(1)^.

Adolescents tend to experience many types of distress, such as concerns over one’s academic performance and future, distress due to school-related issues, bullying, health concerns, general and sexual distress (including gender identity disorder), and distress related to contraception and sexual health. Mental health issues, represented by bullying and truancy, require comprehensive management.

Communication skills are important to maintain resilience against various difficulties. Communication skills and other relationship-building skills include controlling oneself, thinking of others, conveying one’s feelings while considering others’ situations, and taking responsibility for one’s own behaviors. Young people who adapt to difficult situations are likely to have various skills, which include showing consideration to others, having social empathy, understanding others’ feelings, managing one’s emotions and unpleasant experiences, and choosing more effective strategies ^(2)^. Helping with housework and participating in other family members’ important decision-making help them develop self-confidence in starting a family in the future. Thus, these activities positively influence adolescent mental health.

Members of the research group have been providing a session program to increase empathy in expectant couples using role-play and scientifically demonstrating its positive effects on such couples inside and outside Japan. As postpartum depression is also observed among fathers as well as among mothers, mutual emotional support and favorable relationship-building between couple members are thought to be important. The research group created a Japanese version of this program and applied it to married couples during the pregnancy period. The program was favorably evaluated, and it confirmed the feasibility of improving postpartum depression in mothers by promoting empathy in fathers ^(3)^.

The research group also applied the program to university students ^(4)^, in addition to married and expectant couples, revealing that nurturing empathy in adolescents helps promotes their interpersonal relationship-building skills and consequently contributes to their preparedness for parenthood. However, experiences and learning opportunities for males and females to develop parenthood from late young adulthood to the age at first marriage are insufficient at present. Young adulthood is an important preparatory period to acquire parenthood. In this respect, the epidemiological findings of this study on practical education to prepare for parenthood involving young people before pregnancy may be valuable.

The paper published in this journal ^(5)^ examines senior high school students as adolescents and proves the feasibility of more scientifically promoting empathy in young people with waiting list control. Programs focusing on the emotional aspect of empathy are also important to prevent the major problems faced by educational institutions, such as violence and bullying. The program to increase empathy was suggested to be applicable to actual school settings, similarly to the present case.

The program, where participants perform groupwork with same-age peers while visualizing life during pregnancy and after childbirth, and share solutions while improving their own communication skills, may also be effective in terms of life skill acquisition, because it promotes empathy, problem-solving, communication, and interpersonal relationships as life skills to be acquired from adolescence, as stated by the WHO. Furthermore, its effect of raising awareness among young generations preparing for parenthood to play the role of a parent, regardless of their sex, increasing their knowledge of pregnancy, delivery, and parenting, and providing an opportunity for future life planning may be similarly important.

In future studies, it may be necessary to follow-up with participants and confirm the effectiveness of the program, rather than conduct mental assessment at a single point immediately after participation. The present study may have marked significance in implementing and evaluating such a program as part of health education through collaboration with schools, while considering their current situation, where heavy education curricula make it difficult to allocate special class hours for health education. In Japan, which is facing serious problems related to adolescence but has difficulty in disseminating preventive education, these outcomes may provide an important basis for solutions.

## Article Information

### Conflicts of Interest

None
